# Old age and multiple comorbidity are associated with delayed diagnosis of Guillain–Barre syndrome

**DOI:** 10.1038/s41598-022-14184-z

**Published:** 2022-06-15

**Authors:** Sohyeon Kim, Hee Jo Han, Ha Young Shin, Seung Woo Kim

**Affiliations:** Department of Neurology, Yonsei University College of Medicine, 50-1, Yonsei-ro, Seodaemun-gu, Seoul, 03722 South Korea

**Keywords:** Diseases, Neurology

## Abstract

To assess whether older age and presence of comorbidities were associated with a delayed diagnosis of Guillain–Barré syndrome (GBS). The medical records of 140 patients diagnosed with GBS at Severance Hospital from March 2011 to December 2020 were retrospectively reviewed. Comorbidity profiles were assessed using the Charlson comorbidity index (CCI). The age-adjusted CCI (ACCI) score was calculated, which further incorporated the effect of age. Patients were classified into the early diagnosis group (diagnosis duration ≤ 14 days) and late diagnosis group (diagnosis duration > 14 days). Clinical features and comorbidity profiles were compared between the two groups. The cumulative incidence of diagnosis was compared between the low and high ACCI groups. Age was significantly higher in the late diagnosis group (61.8 ± 15.0 years) than in the early diagnosis group (49.1 ± 18.4, *p* = 0.001). The CCI score was higher in the late diagnosis group (≥ 3 in 26.1%) than in the early diagnosis group (≥ 3 in 5.1%, *p* = 0.01). The ACCI score demonstrated a positive correlation with the diagnosis duration (β = 1.636, *p* < 0.001), indicating that the diagnosis was delayed in patients with a higher ACCI score. The duration from onset to diagnosis was longer in the high ACCI group than in the low ACCI group (log-rank test, *p* < 0.001). The diagnosis duration was significantly longer, especially in patients with malignancy and cardiovascular diseases. Delayed diagnosis of GBS is associated with older age and multiple comorbidities. Diagnostic delay was significant in patients with malignancies and cardiovascular diseases. Early suspicion of GBS is required in these patients.

## Introduction

Guillain–Barré syndrome (GBS) is a peripheral nerve disorder known to be the leading cause of acute flaccid paralysis. Although consensus diagnostic guidelines have been suggested^1,2^, the diverse clinical manifestations of GBS challenge physicians in making a timely diagnosis. Clinical courses and outcomes of GBS are highly variable as well, from full recovery to major disabilities such as gait impairment, pain, or even death^3–5^. A correct diagnosis with prompt and adequate therapeutic interventions has been emphasized to achieve a better prognosis and reduce permanent disabilities. Early diagnosis was associated with less residual weakness and lower frequency of respiratory distress, whereas a delayed diagnosis was found to be related to mortality in GBS^4,6^.

Several factors have been suggested to be associated with a delayed diagnosis of GBS. A delayed consultation with a neurologist, an atypical pattern of weakness, and co-existing non-motor symptoms were identified as attributing factors in the diagnostic delay of GBS^6,7^. However, little is known about whether age or the degree of comorbidity is associated with a diagnostic delay of GBS. Moreover, multiple comorbidities are associated with a delayed diagnosis in patients with multiple sclerosis^8^. Among patients with Parkinson’s disease, older age and psychiatric comorbidities were associated with a delayed diagnosis^9^. GBS can occur at any age, including the elderly population^10^, and older patients with GBS are highly likely to have chronic comorbidities^11^. As in other diseases, it is possible that old age and multiple comorbidities could lead to an incorrect diagnosis of GBS. It would be necessary to assess whether age and degree of comorbidity are associated with a delayed diagnosis of GBS, and if an association was found, which comorbidity specifically contributes to the delay.

In the present study, we hypothesized that older age and the presence of comorbidities may contribute to a delayed diagnosis of GBS. We compared the age and degree of comorbidities between patients who were diagnosed within 2 weeks of onset and those diagnosed after 2 weeks. In addition, we compared the cumulative incidence of diagnosis between patients with low comorbidity and a high comorbidity index.

## Methods

### Study populations

The medical records of patients who were diagnosed with GBS at Severance Hospital from March 2011 to December 2020 were retrospectively reviewed. Severance Hospital is a tertiary referral hospital located in the northwestern Seoul where emergency physicians or neurologists first contacts the patients in case GBS is suspected. Of the patients diagnosed with GBS, those 1) whose exact date of symptom was uncertain, and 2) whose comorbidities at the point of enrollment were uncertain were excluded. A total of 149 patients diagnosed with GBS were identified during the study period. After exclusion of nine patients whose exact date of onset or the presence of comorbidities were uncertain, 140 patients were finally included. The included patients were divided into two groups based on the duration from symptom onset to diagnosis. Patients were classified into the early diagnosis group if diagnosis was made at or within 14 days of symptom onset and as late diagnosis group if the diagnosis was made > 14 days after the onset. Diagnosis of GBS after 14 days was considered to be delayed as majority of patients with GBS reach nadir within 2 weeks and it is generally recommended to initiate treatment for GBS within 2 weeks after the onset^1,12,13^. This study was approved by the institutional review board of Severance Hospital.

### Diagnosis and classification of GBS

GBS was diagnosed according to the National Institute of Neurological Disorders and Stroke diagnostic criteria based on the clinical symptoms and laboratory findings^14^. Diagnosis was made promptly at the first evaluation by a neurologist based on the clinical suspicion ahead of electrophysiologic test. Patients were classified into subtypes by the presence or absence of major symptoms, including motor weakness, sensory dysfunction, ataxia, facial paresis, bulbar weakness, pain, and ocular symptoms. Subtypes were categorized according to the criteria suggested by the GBS Classification Group in 2014^2^. GBS subtypes other than the classic GBS were categorized as GBS variants, and subtypes of Miller Fisher syndrome (MFS) other than the classic MFS were classified as MFS variants. Finally, patients were classified into four subtypes: classic GBS, GBS variants, classic MFS, and MFS variants.

### Clinical and electrophysiological assessments

Demographic and clinical information, including date of birth, age at onset, previous medical history of comorbidities, clinical symptoms, neurological examination results, and electrodiagnostic test results, were recorded. The Medical Research Council (MRC) sum score and inflammatory neuropathy cause and treatment (INCAT) scores were also recorded, which have been routinely evaluated in patients with GBS. The MRC sum score is an examiner-based assessment scale for strength, which is calculated by summing MRC scores of 16 muscle groups^15,16^. The MRC sum score ranges from 0 to 80, with a lower score indicating severe weakness. The INCAT disability score is an assessment tool for disability in the arms and legs^17^. A score of 0 represents no weakness, whereas a score of 10 indicates severe weakness. Clinical information and assessment scales data were collected at the time of first evaluation by a neurologist. During nerve conduction study, at least four motor nerves (including the median, ulnar, peroneal, and tibial nerves) and four sensory nerves (including the median, ulnar, sural, and superficial peroneal nerves) were examined. The electrophysiological features were classified as normal, axonal, demyelinating, and indeterminate according to Rajabally’s criteria^18–20^.

### Evaluation of comorbidity status

Comorbidities of the patients were retrospectively investigated by reviewing the medical records drafted by neurologists who interviewed the patient during the initial assessment. The comorbidity status of patients was assessed using the Charlson comorbidity index (CCI) score^21^. The CCI score is the most widely used comorbidity score, which is calculated by summing the score of 17 diagnostic categories of comorbidities, each of which is weighted from a score of 1 to 6 according to the mortality risk. The total score ranges from 0 to 30, with higher scores indicating an increased burden of comorbid conditions. We also calculated the age-adjusted CCI (ACCI) score, which assigns 1 point as a risk value for each decade of age over 40. Besides the CCI and ACCI score, the number of patients having malignancy, cardiovascular, neurologic, endocrinologic and nephrologic disease categorized based on CCI scoring system was analyzed.

### Statistical analysis

We analyzed the data using SPSS Statistics V.26.0. Data are expressed as mean with standard deviations, medians with interquartile ranges, or proportions. Student's t-test, Mann–Whitney U test, and Fisher's exact or χ2 test were performed as appropriate to compare the clinical variables between the two groups. Correlations between diagnostic delay and CCI scores were estimated using linear regression analysis. Kaplan–Meier analysis and log-rank tests were conducted to demonstrate and compare the cumulative incidence of diagnosis between the low and high ACCI groups. Multivariate Cox regression analysis was used to assess clinical variables that had independent effects on diagnostic delay. Statistical significance was set at *p* < 0.05.

### Ethical standards

This retrospective chart review study involving human participants was in accordance with the ethical standards of the institutional and national research committee and with the 1964 Helsinki Declaration and its later amendments or comparable ethical standards. The Human Investigation Committee (IRB) of Severance Hospital approved this study. Informed consent was waived by our institutional ethical board since the research involves no more than minimal risk to participants and does not adversely affect the rights and welfare of them.

## Results

### Overall characteristics of the included patients

A total of 140 patients with GBS were included. The mean age of the patients was 51.2 ± 18.4 years and 78 (55.7%) patients were aged ≥ 50 years. Median duration from onset to first contact to neurologist and that from first contact to hospitalization was 4.0 (Q1– Q3, 2.0–8.0) days and 1 (0–1.0) day, respectively. After contact to neurologist, diagnosis was made after 1 (0–1.0) day and initial nerve conduction studies were performed after 1.5 (1.0–3.0) days.

### Comparison of clinical features between the early and late diagnosis group

Of the 140 patients, 117 (83.6%) were classified into the early diagnosis group and 23 (16.4%) into the late diagnosis group. The median interval from onset to the first contact to neurologist was significantly delayed in late diagnosis group (48.0 [19.0–53.0] days) than in early diagnosis group (4.0 [2.0–6.0] days, *p* < 0.001). In contrast, the duration from first contact to hospitalization and that from the first contact to diagnosis did not significantly differ between groups (*p* = 0.471 and *p* = 0.135, respectively). Demographic, clinical, and electrophysiological characteristics of the patients at the point of initial assessment by the neurologists are compared between the two groups (Table 1). The age at diagnosis was significantly higher in the late diagnosis group (61.8 ± 15.0 years) than in the early diagnosis group (49.1 ± 18.4, *p* = 0.001). The CCI score was significantly different between the two groups (*p* = 0.01). The proportion of patients with a CCI score of 0 was 56.5% in the late diagnosis group and 80.3% in the early diagnosis group. In contrast, 26.1% of the patients in the late diagnosis group had CCI scores ≥ 3, whereas only 5.1% of the early diagnosis group had CCI scores ≥ 3. The proportion of the patients having neurologic and endocrinologic disease was significantly higher in the late diagnosis group (13.0% and 30.4%, respectively) than in the early diagnosis group (0.9% and 10.3%,*p* = 0.014 and *p* = 0.017, respectively). The results that compared the frequency of each comorbidity between the late diagnosis group and early diagnosis group are shown in Supplementary table. With regard to the presenting symptoms, ocular or bulbar symptoms were significantly more frequent in the early diagnosis group (38.5% and 34.2%, respectively) than in the late diagnosis group (8.7% and 13.0%, *p* = 0.006 and *p* = 0.044, respectively). There was no significant difference in the proportion of patients with weakness, sensory changes, ataxia, pain, and facial palsy. There was no difference in the electrophysiological subclassification of GBS, mean INCAT disability score, or MRC sum score between the two groups.

**Table 1:** Comparison of clinical and electrophysiological characteristics of the patients with Guillain–Barre syndrome who were diagnosed within 2 weeks and after 2 weeks from symptom onset.

	Late diagnosis (n = 23)	Early diagnosis (n = 117)	*P*
Age at diagnosis, years	61.8 ± 15.0	49.1 ± 18.4	0.001*
Sex, male	15 (65.2)	68 (58.1)	0.526
**CCI score**			0.01*
0	13 (56.5)	94 (80.3)	
1	3 (13.0)	14 (12.0)	
2	1 (4.3)	3 (2.6)	
≥ 3	6 (26.1)	6 (5.1)	
**Diagnosis**			0.471
Classic GBS	16 (69.6)	62 (53.0)	
GBS variant	4 (17.4)	20 (17.1)	
MFS	1 (4.3)	14 (12.0)	
MFS variant	2 (8.7)	21 (17.9)	
**Presenting symptoms**			
Motor weakness	19 (82.6)	82 (70.1)	0.221
Sensory change	12 (52.2)	61 (52.1)	0.997
Ataxia	3 (13.0)	23 (19.7)	0.568
Pain	7 (30.4)	15 (12.8)	0.055
Ocular symptom	2 (8.7)	45 (38.5)	0.006*
Facial palsy	3 (13.0)	13 (11.1)	0.728
Bulbar weakness	3 (13.0)	40 (34.2)	0.044*
**Electrophysiological subclassification**			0.151
Normal	3 (13.6)	34 (29.8)	
Axonal	9 (40.9)	40 (35.1)	
Demyelinating	5 (22.7)	10 (8.8)	
Indeterminate	5 (22.7)	30 (26.3)	
INCAT disability score	4.3 ± 2.8	4.2 ± 3.2	0.844
MRC sum score	63.0 ± 17.8	64.3 ± 16.8	0.725

Early and late diagnosis are defined by time from symptom onset to confirmation of diagnosis as follows: Early(≤ 14 days) and late(≥ 15 days).

CCI, Charlson Comorbidity Index; GBS, Guillain–Barré syndrome; MFS, Miller Fisher syndrome; INCAT, Inflammatory Neuropathy Cause and Treatment; MRC, Medical Research Council.

*p < 0.05.

### Association between the diagnostic delay and ACCI score

**Figure 1:**
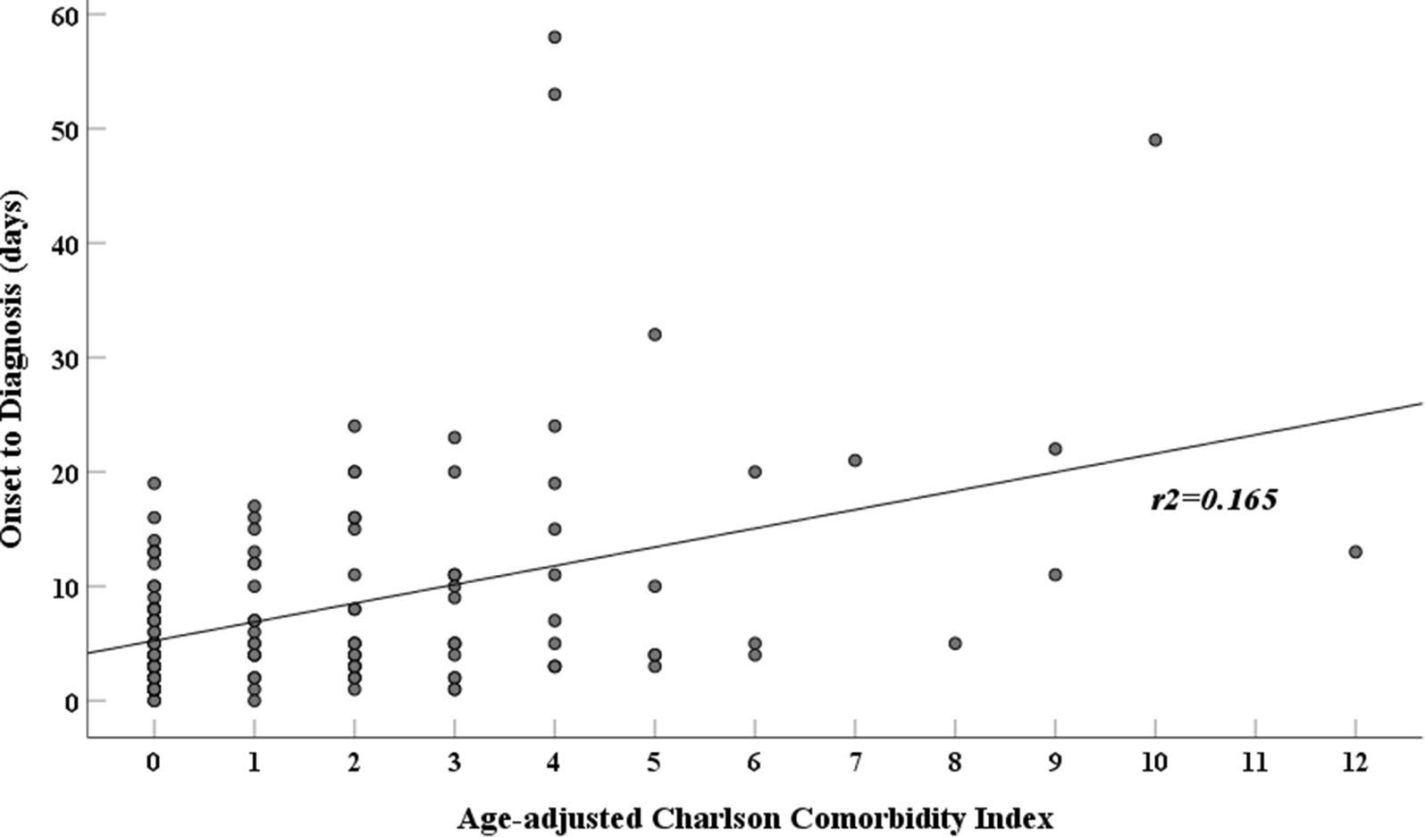
Correlation between age-adjusted Chralson Comorbidity Index score and duration from symptom onset to diagnosis of Guillain–Barre syndrome.

As the patients in the late diagnosis group were older and had higher CCI scores than those in the early diagnosis group, the ACCI score, which integrates the degree of comorbidity and age, was calculated. The mean ACCI score was significantly higher in the late diagnosis group (3.4 ± 2.6) than in the early diagnosis group (1.5 ± 2.1, *p* < 0.001). We further analyzed the association between the ACCI score and the duration from symptom onset to diagnosis. The ACCI score demonstrated a positive correlation with the duration from symptom onset to diagnosis (β = 1.636, *p* < 0.001, Fig. 1), indicating that the diagnosis of GBS was delayed in patients with a higher ACCI score.

### Difference in the diagnostic delay between low and high age-adjusted comorbidity group

**Figure 2:**
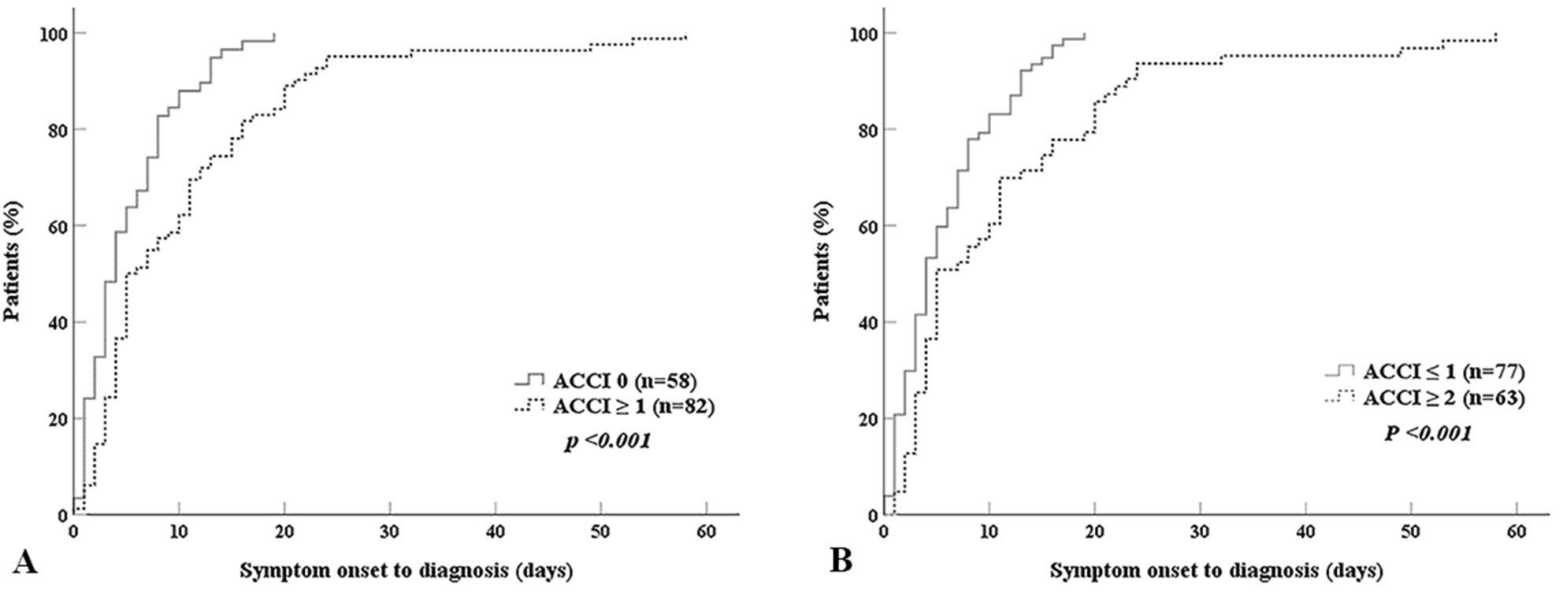
Cumulative incidence curves for diagnosis of Guillain–Barre syndrome after symptom onset are compared between the patients with (**A**) age-adjusted Charlson Comorbidity Index (ACCI) of 0 and ACCI ≥ 1 and (**B**) ACCI ≤ 1 and ACCI ≥ 2.

Patients were divided into those with an ACCI score of 0 (low ACCI score group) and those with ACCI score ≥ 1 (high ACCI score group) to compare the cumulative incidence of diagnosis based on the ACCI score. Fifty-eight (41.4%) patients had ACCI scores of 0, and 82 (58.6%) patients had an ACCI score of 1 or higher. Cumulative incidence curves for the diagnosis of GBS in patients with low ACCI scores and high ACCI score groups are displayed in Fig. 2A. The median duration from the onset of GBS to diagnosis was 4.0 days in the low ACCI score group, whereas the duration was 5.5 days in the high ACCI score group. The duration from onset to diagnosis was significantly longer in the high ACCI score group than in the low ACCI score group (log-rank test, *p* < 0.001). The high ACCI score group still had a 2.08-fold (hazard ratio = 2.08, 95% confidence interval [CI] 1.45–3.00) higher chance of delayed diagnosis than the low ACCI score group after adjusting for the presence of ocular and/or bulbar symptoms. Similar results were observed when the patients were classified into those with ACCI scores of 0 or 1 (low ACCI score group) and those with ACCI ≥ 2 (high ACCI score group, Fig. 2B). Time to diagnosis was significantly longer in the high ACCI score group than in the low ACCI score group (log-rank test, *p* < 0.001).

### Diagnostic delay in each comorbidity

The duration from the onset of GBS to diagnosis was compared between patients with and without each type of comorbidity (Table 2). Median duration from the onset to diagnosis was significantly longer in patients with malignancy and with cardiovascular diseases (10.5 [5.0–18.3] and 11.0 [4.0–49.0] days, respectively) than those without the disease (5.0 [3.0–10.8] and 5.0 [3.0–11.0], *p* = 0.04 and *p* = 0.038, respectively). We further analyzed the reason for diagnostic delay in three patients with malignancy and three patients with cardiovascular disease in whom the duration of diagnosis exceeded 2 weeks. In patients with malignancy, the evaluation for metastasis delayed the diagnosis (n = 3, 100%). In patients with cardiovascular disease, the cause of late diagnosis of GBS was the evaluation of cerebrovascular disease (n = 3, 100%). There was no significant difference in the duration from onset to diagnosis between patients with and without other types of comorbidities including neurologic, endocrinologic and nephrologic disorders. We further compared the age between the patients with and without each comorbidity to assess whether the diagnostic delay was associated with the difference in age. Patients with malignancy or endocrinologic disease were found to be older (70.0 [60.5–75.5] and 67.0 [59.0–74.0] years, respectively) than those who did not have these comorbidities (51.5 [33.3–64.8] and 51.0 [32.0–64.0] years, *p* = 0.002 and *p* < 0.001; respectively).

**Table 2:** Comparison of duration from symptom onset to diagnosis in patients with Guillain–Barre syndrome classified by particular comorbidity categories.

Comorbidity category	Unaffected, median (IQR)	Affected, median (IQR)	*P*
Malignancy	5.0 (3.0–10.8), n = 128	10.5 (5.0–18.3), n = 12	0.04*
Cardiovascular	5.0 (3.0–11.0), n = 133	11.0 (4.0–49.0), n = 7	0.038*
Neurologic	5.0 (3.0–11.0), n = 136	19.5 (6.0–45.8), n = 4	0.057
Endocrinologic	5.0 (3.0–10.5), n = 121	5.0 (3.0–21.0), n = 19	0.137
Nephrologic	5.0 (3.0–11.0), n = 135	12.0 (4.0–17.0), n = 5	0.197

IQR, Interquartile ranges.

*p < 0.05.

## Discussion

A delayed diagnosis of GBS is associated with older age and high comorbidity profiles. The cumulative incidence curve for the diagnosis showed that diagnosis was significantly delayed among patients aged ≥ 50 years or those with CCI scores ≥ 1 compared to younger patients without comorbidity. Among the comorbidity categories, diagnostic delay was significant in patients with malignancy and cardiovascular disease. Considering that the MRC sum score, INCAT disability score, and electrophysiological subclassification of GBS did not present significant differences between groups, it can be inferred that delay in diagnosis was less likely to be associated with the severity of the disease or with the lack of evidence of GBS in nerve conduction studies. Early suspicion of GBS is required in older patients and those with multiple comorbidities displaying subacute progressive weakness.

The association between diagnostic delay and comorbidities in neurological diseases has been suggested in previous studies. Katyal et al. recently showed that the time to diagnosis of GBS is longer in patients with coexisting neurological conditions than in those without^7^. The present study demonstrated that not only neurological disease but also other comorbidities, including malignancy and cardiovascular disease, should be taken into consideration. The association between comorbidity and late diagnosis was similarly observed in multiple sclerosis, Parkinson’s disease, and malignancy^8,9,22,23^. A recent study similarly stated that the patients with comorbidities took longer for the diagnosis of GBS as well^24^. According to our results, it was reasonable to infer that late consultation to the neurologist or delayed hospital visit made difference in the diagnosis duration. One of the hypotheses that explain this behavior is the “competing demand hypothesis,” which suggests that pre-existing health conditions lead to indifference to newly developed symptoms and that physicians are likely to attribute new symptoms to known diseases^25^. In the present study, this tendency was observed as newly developed weakness in patients with malignancy being overlooked or understood as cancer-related fatigue or result of cancer metastasis. Similarly, physicians were likely to prioritize the possibility of cerebrovascular disease in patients with underlying cardiovascular diseases. To avoid misdiagnosis, it is crucial to obtain a meticulous history regarding the antecedent events, presentation of neurological symptoms, and progression.

Old age at diagnosis was another factor associated with a delayed diagnosis. Delayed diagnosis of GBS in patients with malignancy may be partially explained by the older age of the patients with malignancy than in those without. Old age has been shown to be associated with a delayed diagnosis across various neurological or non-neurological diseases, including Parkinson’s disease, pulmonary hypertension, and tuberculosis infection^9,26,27^. It has been suggested that access to medical evaluation is prompted when a patient perceives symptoms more seriously, while the presentation is delayed in patients with lower socioeconomic status and more comorbidities^23,25^. It is suggested that older people lack recognition of the significance of symptoms and tend to have more comorbidities and belong to the lower socioeconomic status than younger people^28–32^. This may explain the diagnostic delay in elderly patients. However, age was not found to be associated with the diagnostic delay in a previous study that also analyzed the risk factors associated with the diagnostic delay of GBS^7^. The previous study was based on a relatively small number of patients and compared the duration of diagnosis between patients aged < 50 years and those aged ≥ 50 years, and this may have led to different results. In another study, duration of symptom was rather shorter in GBS patients aged ≥ 60 years than those aged < 60 years^33^. However, elderly patients with GBS more frequently had cranial nerve dysfunction and had higher disability than adult patients, and this may have alarmed the physicians and contributed to early diagnosis. As GBS in the elderly is associated with severe illness with late recovery, longer hospitalization, and higher incidence of pneumonia^11,33–36^, early diagnosis of GBS with rapid therapeutic interventions should be emphasized, particularly in older populations.

Bulbar and ocular symptoms were associated with an early diagnosis of GBS in the present study. This is in line with the result that, although not significant, MFS or its variant was more common in the early diagnosis group, whereas classic GBS was more frequent in the late diagnosis group. This result indicates that GBS presenting with only limb weakness and without cranial nerve dysfunction could be overlooked by first-contact physicians, whereas the presence of cranial nerve involvement may lead to immediate consultation with a neurologist, which is a known factor associated with the early diagnosis of GBS^6^. In terms of other symptoms, a previous study suggested that pain contributes to a delayed diagnosis^6^. Although not significant, the present study showed a similar tendency that patients presenting with pain tended to be diagnosed late. Pain is reported in approximately one-third of patients with GBS and can be severe enough to affect their quality of life^37^. However, pain is often poorly recognized and is undertreated^38^. It should be understood that pain could be one of the initial symptoms of GBS.

Our study has several limitations. First, it is uncertain whether our findings can be generalized into a larger population size since we recruited patients from a single center. In addition, the number of patients with each type of comorbidity was relatively small; thus, the association between specific comorbidities and delayed diagnosis of GBS cannot be generalized. Second, considerations are needed in interpretation of the results as other confounding factors for diagnostic delay, including geographical proximity, socioeconomic status, and expertise of the first contact physician, were not meticulously clarified in the study. In addition, the consequences of late diagnosis could not be accurately evaluated due to the retrospective nature of the study. Thus, it cannot be determined whether a delayed diagnosis in older patients and those with heavy comorbidities is associated with poor prognosis based on the present result. Although the present study lacks a prognostic aspect, it can be expected that late diagnosis could worsen clinical outcomes, as previous studies have consistently elucidated^3,4^. Further studies based on larger cohorts are required which can comprehensively assess regional and ethnic diversity, socioeconomic factors, and prognostic aspects in detail.

In conclusion, older age and multiple comorbidities were found to be associated with the delay in the diagnosis of GBS. Physicians should consider the possibility of GBS in elderly patients with multiple comorbidities.

## Supplementary Information

Supplementary Tables.

## Data Availability

The datasets used and/or analyzed during the current study available from the corresponding author on reasonable request.
