# Supplementary material for: Old age and multiple comorbidity are associated with delayed diagnosis of Guillain–Barre syndrome
Source: Sci Rep. 2022 Jun 15;12:9913. doi: 10.1038/s41598-022-14184-z (PMC9200811; doi:10.1038/s41598-022-14184-z)
Supplement: Supplementary file 1 — Supplementary Tables. [file 41598_2022_14184_MOESM1_ESM.docx]

**Supplementary Table. Frequency of each comorbidity compared in early and late diagnosis groups**

| Comorbidity category | Late diagnosis  (n=23) | Early diagnosis (n=117) | *P* |
| --- | --- | --- | --- |
| Malignancy, n(%) | 3 (13.0) | 9 (7.7) | 0.417 |
| Cardiovascular | 3 (13.0) | 4 (3.4) | 0.087 |
| Neurologic | 3 (13.0) | 1 (0.9) | 0.014* |
| Endocrinologic | 7 (30.4) | 12 (10.3) | 0.017* |
| Nephrologic | 2 (8.7) | 3 (2.6) | 0.189 |

*p<0.05
